# O.4.2-6 The complexity of physical activity and alcohol consumption in adolescents aged 13-16 years: cross-sectional analysis of Avon Longitudinal Study of Parents and Children data (ALSPAC)

**DOI:** 10.1093/eurpub/ckad133.181

**Published:** 2023-09-11

**Authors:** Saphsa Codling, Thomas Phillips, Colin R Martin, Chao Huang, Lesley Smith

**Affiliations:** University of Hull, UK; University of Hull, UK; Institute for Health and Wellbeing, University of Suffolk, Ipswich, UK; Hull York Medical School, University of Hull, UK; s.codling-2019@hull.ac.uk; University of Hull, UK

## Abstract

**Purpose:**

*A recent review indicated that physical activity (PA), facilitated by organisations/clubs, may reduce alcohol consumption in early to mid-adolescence. Our study aims were to examine these factors, and identify how health determinants may influence association*.

**Methods:**

Cross-sectional secondary data analysis using UK cohort data from ALSPAC. Ages and sample-size: Time-point (TP) 1: 13-14 (n = 1824), TP2: 15-16 (n = 1334). Variables: minutes per day spent in moderate to vigorous PA > =3 days of accelerometer wear. Ancillary PA variables: Club-type (CT); and frequency of attending club (FAC) collected age 15 only. Risk of alcohol-related harm (RARH) categorised as: no current risk, increasing risk, and at risk (AR). Ordinal regression was conducted at each TP, using PA as a covariate. Explanatory variables (EVs): Psychosocial health (PSH) - Cluster membership numbers generated through K-means cluster analysis (uniquely represented at each TP); Socioeconomic status (SES); Educational attainment; BMI; Smoking status, and Gender. EVs entered into regression model if preliminary X^2^ tests achieved *p*<.10.

**Results:**

Regression showed a positive association between PA and RARH at both TPs. Odds of being AR at TP1 were 1.31 greater for each 30 minute increase in PA (95%CI 1.10-1.57; *p*<0.002). At TP2: OR 1.24 (95%CI 1.01, 1.52; *p=.*036). **TP1:** CT (age 15) was not statistically significant at age 13. However, ‘sports club only’ (SCO), had greatest RARH (OR 1.04; 95%CI .806, 1.35; *p=.*753) vs. no CT. All EVs retained statistical significance at *p*<.05, excepting IDACI (*p = *.854) and gender (*p = *.917). **TP2**: Although CT was not statistically significant, SCO had the greatest RARH (OR 1.22; 95%CI .798, 1.86; *p*=.360). RARH was reduced if attending club most evenings vs. no attendance (OR .796; 95%CI .499,1.27; *p=.388*). Statistically significant EVs were PSH (OR 4.27; 95%CI 3.32, 5.51; *p*<.001) and smoking (OR 5.39; 95%CI 3.99, 7.29; *p*<.001). Model fit χ2 331.010, df 11, *p*<0.001. Pearson .455. Deviance .218. Nagelkerke .251. *-2LL* test of parallel lines with significance .321.

**Conclusions:**

The relationship between PA and alcohol consumption is complex. While facilitated PA can provide many benefits for adolescents, potential unwanted consequences may be an increase in risk-behaviours like alcohol consumption. Further research is needed for greater comprehension of this association.

